# The Cost of Direct Care Workforce Instability: Economic and Health Impacts of High Turnover in Pennsylvania

**DOI:** 10.1093/geroni/igaf122.1837

**Published:** 2025-12-31

**Authors:** Sarah Aviña, Amanda Kreider, Howard Degenholtz

**Affiliations:** University of Pittsburgh, Pittsburgh, Pennsylvania, United States; University of Pittsburgh, Pittsburgh, Pennsylvania, United States; University of Pittsburgh, Pittsburgh, Pennsylvania, United States

## Abstract

Pennsylvania’s direct care workforce crisis is intensifying, with annual turnover rates between 44–65% and an estimated shortfall of over 37,000 workers by 2026. While workforce shortages in long-term services and supports (LTSS) are well-documented, the financial burden of turnover remains underexplored. This study quantifies the economic and health costs of workforce instability, focusing on avoidable emergency department (ED) visits, hospitalizations, and long-term care admissions linked to staffing shortages. Using longitudinal workforce and wage data from the Bureau of Labor Statistics (BLS), PHI, and the Pennsylvania Department of Human Services (DHS) 2025-2026 Blue Book, along with Pennsylvania Medicaid data from 2024-2025, we assess turnover trends and cost implications. We find that direct care workforce shortages are associated with a 15-20% increase in preventable ED visits and a 12-18% increase in hospitalizations, adding as much as $75 million in excess Medicaid expenditures annually. Comparing Pennsylvania’s LTSS workforce trends against national data suggests that that the state’s crisis is disproportionately severe, surpassing the national average by 10%, with rural and low-income populations disproportionately affected. Findings indicate that each direct care worker turnover costs providers an estimated $4,200, leading to millions in annual financial losses and service instability. Policy recommendations include retention-based Medicaid reimbursements, expansion of direct care worker roles in interdisciplinary care teams, and pipeline initiatives. This study underscores the need for evidence-based policy reforms to address workforce instability and prevent excess healthcare costs in the Medicaid LTSS system.

